# Correlation of three immunohistochemically detected markers of neuroendocrine differentiation with clinical predictors of disease progression in prostate cancer

**DOI:** 10.1186/1471-2490-8-21

**Published:** 2008-12-30

**Authors:** M Hammad Ather, Farhat Abbas, Nuzhat Faruqui, Mohammad Israr, Shahid Pervez

**Affiliations:** 1 Department of Surgery, Aga Khan University, Karachi, Pakistan; 2 Department of Pathology, Aga Khan University, Karachi, Pakistan

## Abstract

**Background:**

The importance of immuno-histological detection of neuroendocrine differentiation in prostatic adenocarcinoma with respect to disease at presentation and Gleason grade is gaining acceptance. There is limited literature on the relative significance of three commonly used markers of NE differentiation i.e. Chromogranin A (CgA), Neuron specific enolase (NSE) and Synaptophysin (Syn). In the current work we have assessed the correlation of immuno-histological detection of neuroendocrine differentiation in prostatic adenocarcinoma with respect to disease at presentation and Gleason grade and to determine the relative value of various markers.

**Materials and methods:**

Consecutive samples of malignant prostatic specimens (Transurethral resection of prostate or radical retropubic prostatectomy) from 84 patients between January 1991 and December 1998 were evaluated by immunohistochemical staining (PAP technique) using selected neuroendocrine tumor markers i.e. Chromogranin A (CgA), Neuron specific enolase (NSE), and Synaptophysin (Syn). According to the stage at diagnosis, patients were divided into three groups. Group (i) included patients who had organ confined disease, group (ii) included patients with locally invasive disease, and group (iii) with distant metastasis. NE expression was correlated with Gleason sum and clinical stage at presentation and analyzed using Chi-Square test and one way ANNOVA.

**Results:**

The mean age of the patients was 70 ± 9.2 years. Group I had 14 patients, group II had 31 patients and group III had 39 patients. CgA was detected in 33 cases, Syn in 8 cases, and NSE in 44 cases. Expression of CgA was seen in 7% of group I, 37% in group II and 35% of group III patients (p 0.059). CgA (p 0.024) and NSE (p 0.006) had a significantly higher expression with worsening Gleason grade.

**Conclusion:**

CgA has a better correlation with disease at presentation than other markers used. Both NSE and CgA had increasing expression with worsening histological grade this correlation has a potential for use as a prognostic indicator. Limitations in the current work included small number and retrospective nature of work. The findings of this work needs validation in a larger cohort.

## Background

Prostate cancer is the most commonly diagnosed malignancy in men in the United States with an estimated 218,890 cases diagnosed in the year 2007 and estimated death of 27,050 [1]. Among men, cancers of the prostate, lung and bronchus, and colon and rectum account for about 54% of all newly diagnosed cancers, prostate cancer accounts for about 33% of cases in men [1]. Prostate cancer incidence rates continued to increase, although at a slower rate than those reported for the early 1990s and before. Based on cases diagnosed between 1995 and 2001, an estimated 91% of these new cases of prostate cancer are expected to be diagnosed at local or regional stages, for which 5-year relative survival approaches 100% [1]. However, it is noteworthy that individual cancers show substantial variation in its outcome. The variable biological potential of these tumors makes it important to stage the disease. The various prognostic indicators include clinical staging, serum PSA, % biopsy core involved and histological grade. The histological grade correlates both with local invasiveness and the metastatic potential. In a subset of both localized and locally advanced cancers, the existing markers, however, are often unable to differentiate poor from good outcome cancers. On these grounds, it is important to establish validated prognostic indicators that could help physicians in tailoring treatment for individual patients.

Neuroendocrine (NE) differentiation in PC has received increasing attention in the recent years due to prognostic and therapeutic implications. The term NE differentiation in prostatic carcinoma includes tumors composed exclusively of NE cells (the rare and aggressive small cell carcinoma and carcinoid/carcinoid like tumor) or, more commonly, conventional prostatic adenocarcinoma with focal NE differentiation [2]. The prognostic importance of focal neuroendocrine differentiation in PC is controversial, but current evidence suggests that it has an influence on prognosis related to hormone resistant tumours or a role in the conversion to a hormone resistant phenotype [3].

Various neuroendocrine markers like Chromogranin A (CgA), synaptophysin (Syn), neuron specific enolase (NSE), β HCG have been studied. However, CgA appears to be the best overall tissue and serum marker [4]. In the current study we have investigated the importance of immuno-histological detection of neuroendocrine differentiation in prostatic adenocarcinoma with respect to disease at presentation and Gleason grade. In addition the relative significance of three markers of NE differentiation i.e. CgA, NSE and Syn is also correlated with stage and grade of disease.

## Methods

This study was conducted following Aga Khan University's ethical review committee (ERC) clearance, in view of the nature of study; ERC waived the requirement for informed consent. Consecutive malignant primary prostatic specimens, were obtained from 84 patients by either trans-urethral resection of prostate (n = 69 patients) for urinary obstruction or from radical retro-pubic prostatectomy (n = 15 patients) between January 1991 and December 1998. These tissue specimens were taken from the archived records of the department of pathology. The age ranged from 52–93 years (mean 70 + 9.2 years). Sections were stained for H & E as well as for Chromogranin A, Synaptophysin and NSE (DAKO), Glostrup, by immuno histochemistry using PAP technique. The methods have also been previously described in details [5]. Clinical staging was done using the TNM system. For patients who had radical retropubic prostatectomy, the T and N stage were pathological and for patients who only had TURP was radiological.

Briefly, 3 μm thick tissue sections were cut and mounted on poly-L-lysine (sigma) coated slides. Sections were deparaffinized in xylene and re-hydrated through graded alcohol series followed by water. Sections were washed with water followed by Phosphate buffer saline (PBS) rinse. Endogenous peroxidase in the sections was blocked for 30 minutes with 0.3% H_2 _0_2 _in methanol. Sections were washed with PBS. All sections were treated with Normal Swine serum (NSS) prediluted 1:10 in PBS for 5 minutes.

The sections were then incubated with the primary antibodies pre diluted appropriately in NSS for 90 minutes at room temperature. Slides were then washed with PBS and incubated with peroxidase-conjugated swine anti rabbit secondary antibody (DAKO) at a dilution of 1:150 for 45 minutes at room temperature. This was followed by inoculation with PAP complex. 3, 3'-diaminobenzidine (DAB) was used as a final Chromogen. Harris Haemtoxylin was used as a counter nuclear stain. Positive controls were used with all batches of IHC staining. Same case by omitting the primary antibody was used as a negative control with each staining procedure. Histological grading, the Gleason system, was used for grading of the cancer specimens; a senior histopathologist (SP) blinded of previous Gleason grading and clinical course performed rescoring. A consensus in departmental consultation conference was achieved in case of any discrepancy. Based upon the Gleason score patients were divided into three groups i.e. well differentiated (Gleason sum 2–4), moderately differentiated (Gleason sum 5–7) and poorly differentiated (Gleason sum 8–10). To study correlation and determine the p value Student t was applied. Statistical significance was examined by Mann-Whitney *U*-test, Student's *t*-test, Kruskal-Wallis test, the log-rank test, and Simple regression. A *P*-value below 0.05 was considered to be significant.

## Results

During the period of 1991–1998 there were 84 patients with histological specimens from TURP and RRP. The mean age was 70 ± 9.3 years. Majority of patients had either locally invasive (37%) or metastatic disease (45%) and only 18% had organ confined disease. At a median follow up of 8.4 ± 3.5 years 54% (n 45) were dead; of the surviving 46% (n 39) 21 patients (25%) had metastatic disease. There is a statistically significant difference in the development of metastases, overall and cause specific survival between groups with and without CgA staining.

According to the TNM classification 35% (n = 29) had stage T1, 32% (n = 29) stage T2, 25% (n = 21) stage T3 and 6% (n = 5) stage T4 disease according to the TNM classification. Based upon the stage of the disease patients were divided into three groups i.e. organ confined (T1-2), locally invasive (T3-4 and N1) and metastatic (M1) cancer. Staining for NE marker (CgA) was seen in 39%, NSE in 52% and Syn in only 10%. The % expression of the three markers in the organ confined, locally advanced and metastatic disease is shown in table 1. It is note worthy that tumors with negative CgA staining were picked up in the early stage with minimal or organ confined disease; the positive results were obtained for locally advanced and metastatic disease (p 0.059). The correlation is not statistically significant but only shows a trend towards the significance. The correlation between Gleason sum and % expression of the three NE markers is shown in table 1. It indicates modest (for CgA) to significant (NSE) relationship between the extent of NE differentiation and Gleason score. In table 2, significance of CgA and NSE is correlated with overall, cancer specific survival and development of metastatic disease. There was no significant correlation in CgA, NSE and SYN expression with the androgen withdrawal status.

**Table 1:** Clinical characteristics' of patients undergoing immunohistochemical analysis and the difference in various parameters between IHC expressed (+ve) and those that did not express IHC markers (-ve)

	**CgA+ve n = 30**	**CgA -ve n = 54**	**p value**	**NSE +ve n = 44**	**NSE -ve n = 40**	**p value**	**SYN +ve n = 8**	**SYN -ve n = 76**	**p value**
**Age (median ±)**	73.2 ± 6.3	69.1 ± 7.4	0.22	70.3 ± 7.4	68.4 ± 7.1	0.33	70.9 ± 9	71.2 ± 8.1	0.20

**PSA mean (ng/ml)**	432	310	0.10	606	444	0.10	112	412	0.01

**Clinical stage **Organ Confined	5	12	0.03	12	5	0.02	2	15	0.001

Locally Advanced	8	27	0.02	12	23	0.05	3	32	0.001

Metastatic	17	26	0.10	20	23	0.32	3	40	0.001

**Gleason sum (mean)**	6.9	6.1	0.05	6.1	7.2	0.04	7	6.3	0.22

CgA: Chromogranin A

NSE: Neuron specific enolase

SYN: Synaptophysin

**Table 2:** Impact of immunohistochemical staining, IHC expressed (+ve) and those that did not express IHC markers (-ve), for Chromogranin A and neuron specific enolase on overall and cause specific survival

	**CgA+ve**	**CgA-ve**	**p-value**	**NSE +ve**	**NSE -ve**	**p value**
**Overall expression (n, %)**	30, 36%	54, 64%	0.005	44,48%	40,52%	0.22

**Overall survival (n, %)**	5, 17%	45, 83%	0.001	24,55%	26,65%	0.32

**Cancer specific survival (n, %)**	4, 13%	41, 76%	0.004	21,48%	24,60%	0.30

**Metastatic disease (n, %)**	18, 60%	17, 32%	0.002	19,43%	16,40%	0.24

CgA: Chromogranin A

NSE: Neuron specific enolase

## Discussion and conclusion

In the present work we have shown NE differentiation in conventional prostate adenocarcinoma and assessed the relationship of the extent of NE status to the commonly recognized prognostic variables. We have also tried to evaluate the relative significance of immunohistochemically detected expression of three markers viz. CgA, SYN, and NSE.

Prostate cancer is a leading cause of morbidity and mortality in men, accounting for 33% of all new cases of cancer and 14% of deaths from cancer [1]. Despite considerable advances in our ability to detect and treat PC, there have been no significant corresponding decreases in morbidity and mortality [6]. The therapeutic aim is to tailor the approach to the clinical, morphological, and molecular features of each patient. Many of the clinically important predictive factors in PC are still derived from a pathologist's examination of tissue specimens using light microscopy, but the challenge of assembling the information is such that the use of artificial neural networks is expected to improve accuracy in diagnosis, staging, and treatment outcomes for PC [3,7]. PC may show divergent differentiation towards a neuroendocrine phenotype in the form of neuroendocrine small cell carcinoma or carcinoid-like tumours [8]. Much more common, however, is focal neuroendocrine differentiation in PC, which may be pronounced in about 10% of carcinomas. The prognostic importance of focal neuroendocrine differentiation in PC is controversial, but current evidence suggests that it has an influence on prognosis related to hormone resistant tumours or a role in the conversion to a hormone resistant phenotype [3]. However, we did not find a significant correlation in CgA, NSE and SYN expression with the androgen withdrawal status. CgA appears to be the best overall tissue and serum marker of neuroendocrine differentiation, and thus serum CgA concentrations may be useful in assessing the emergence or progression of hormone resistant cancer [8]. Recently Kamiya et al noted that CgA had a stronger relationship between serum levels and IHC positivity in contrast to NSE, suggesting clinical usefulness as a tumor marker in predicting the extent of neuroendocrine differentiation in prostate cancer [9].

In our series CgA expression was seen in 31%, Synaptophysin in only 8% and NSE in 45% cases. Only CgA expression was significantly correlated with the clinical stage of the disease, whereas both CgA and NSE correlated with the grade. The significant relationship between tumor grade and NE differentiation was found in some studies [10-12] whereas other investigators failed to confirm [13,14]. In the present study there is a significant correlation between rising Gleason sum and expression of both CgA (p 0.024) and NSE (p 0.006). The relationship between stage of the disease and NE expression was noted only for CgA, whereas for both the other markers it was not statistically significant.

We also compared CgA expression with established NE markers used widely to identify NE cells and NED in the prostate. CgA, a secreted acidic product of prostate NE cells, is a widely accepted and specific marker of both NE cell populations and NED differentiation [15,16]. NSE is another classical NE marker, but lacks some specificity compared to CgA. Serum concentrations of CgA and NSE can be monitored as a potential prognostic factor in PCa [17-19]. In tumor tissue, we found a statistically significant correlation of CgA to NSE expression. Synaptophysin, a presynaptic vesicle glycoprotein, is expressed in virtually all cells of well-differentiated prostate NE tumors (NET, carcinoid), neuroendocrine carcinomas (NEC), and in poorly differentiated carcinomas including small cell carcinoma (SCC), all being very rare clinic entities (0.2–1%) [20]. However, SYN has a lower specificity for NE cells than CgA and may stain positive in non-NE tumors [21]. CgA seems to stain prostate NE cell populations more homogenously than SYN.

Limitation in current work included small number of patients which makes it to difficult to draw definite conclusions concerning the predictive value of various markers of NE differentiation in relation to disease progression and survival. However, the trends are interesting and warrant further work in a larger cohort of patients.

In conclusion this study further supports the theory that focal NE differentiation within classical prostate carcinoma is predictive of poor prognosis, as it correlates with Gleason sum and clinical stage of the disease. In our study, CgA was the best predictor of NE differentiation as it correlated better than the other two markers examined, both with stage and grade of the disease. Given the relatively small sample size of this study, these correlative findings suggest that the prognostic impact of these markers merits further investigation in a larger cohort

## Competing interests

The authors declare that they have no competing interests.

## Authors' contributions

MHA conceived the idea, designed methodology, wrote the grant proposal, coordinated in data collection, analyzed results and wrote the manuscript. FA helped in writing grant proposal, helped in designing methodology, and helped in writing the manuscript. NF did data collection, data analysis, and helped in writing the manuscript. MI performed pathological assessment of specimen, IHC analysis, re Gleason staging etc. SP study design, methodology, review the manuscript and supervised pathology part of the study including IHC, Hand E and Gleason re staging.

## Funding

Authors received a grant from the University research council seed money grant form Aga Khan University to conduct the study. Authors received no other grant for study design; in the collection, analysis, and interpretation of data; in the writing of the manuscript; and in the decision to submit the manuscript for publication.

## Pre-publication history

The pre-publication history for this paper can be accessed here:


